# Serum levels of Trimethylamine-N-oxide in patients with ischemic stroke

**DOI:** 10.1042/BSR20190515

**Published:** 2019-06-18

**Authors:** Maimaiti Rexidamu, Hongmei Li, Haiyan Jin, Jiankang Huang

**Affiliations:** 1 Department of Neurology, Kashgar Prefecture Second People’s Hospital, Kashgar, Xinjiang Uygur Autonomous Region, China; 2 Department of Neurology, Shanghai Jiao Tong University Affiliated Sixth People’s Hospital, Shanghai, China

**Keywords:** ischemic stroke, risk, severity, Trimethylamine-N-oxide

## Abstract

**Objective**: Accumulating evidence suggests that Trimethylamine-N-oxide (TMAO), a gut microbial metabolite, is implicated in the pathogenesis of many cardiovascular diseases. The aim of the present study was to investigate the serum levels of TMAO in Chinese patients with ischemic stroke.

**Method**: In the present study, 255 consecutive patients with first-ever acute ischemic stroke and 255 age and gender-matched healthy volunteers were included for testing serum TMAO. Stroke severity was determined by the NIH Stroke Scale (NIHSS). The stroke severity was dichotomized as minor (NIHSS ≤ 5) and moderate-to-high clinical severity (NIHSS > 6).

**Results**: The serum levels of TMAO in stroke ranged from 0.5 to 18.3 μM, with a median value of 5.8 (interquartile range (IQR), 3.3–10.0) μM, which was higher than in those controls (3.9; IQR, 2.6–6.1 μM). The median level of TMAO in those patients was significantly lower than in those moderate-to-high stroke patients (4.1 μM [IQR, 2.8–6.2] vs. 9.1 μM [5.1–11.0]; *P*<0.001). In univariate and multivariable models, the unadjusted risk of moderate-to-high stroke was increased by 31% (odds ratio (OR) = 1.31 [95% confidence interval (CI): 1.21–1.42], *P*<0.001) and 22% (OR = 1.22; 95% CI = 1.08–1.32; *P*<0.001), when TMAO was increased each by 1 μM. Based on the receiver operating characteristic (ROC) curve, the optimal cut-off value of serum level of TMAO as an indicator for screening of moderate-to-high stroke was estimated to be 6.6 μM, which yielded a sensitivity of 69.3 % and a specificity of 79.0%, with the area under the curve at 0.750 (95% CI, 0.687–0.812).

**Conclusions**: Higher TMAO levels were associated with increased risk of first ischemic stroke and worse neurological deficit in Chinese patients.

## Introduction

Recent studies indicate a pathophysiological contribution of gut microbiota to cardiometabolic diseases with mechanistic links to gut microbial choline metabolism [1]. Trimethylamine-N-oxide (TMAO) is a circulating organic compound produced by the metabolism of dietary l-carnitine and choline, which was recently found to directly induce atherosclerosis in rodents [2–3]. Both l-carnitine and choline are metabolized by intestinal bacteria trimethylamine, a metabolite which is absorbed from the intestine and subsequently oxidized via hepatic flavin monooxygenase enzymes to form TMAO [4].

Previous study had found that TMAO was associated with mortality and hospitalization for cardiorenal disorders, such as heart failure [5], atrial fibrillation [6], acute myocardial infarction [7] and chronic kidney disease [8]. One study in a community-based general population showed that serum TMAO could improve the prediction of future cardiovascular diseases [9], while one study in diabetes found that higher fasting plasma concentrations of TMAO portend higher major adverse cardiac events and mortality risks independent of traditional risk factors, renal function, and relationship to glycemic control [1].

Interestingly, TMAO has been reported to increase thrombotic risk with induced platelet hyperreactivity as the underlying mechanism [10], suggesting increased TMAO to a potential risk for acute ischemic events. Moreover, elevated TMAO levels have been shown to predict a future risk of major adverse cardiac events (MACE), an increased prevalence of cardiovascular disease, and have shown a relationship with the number of diseased coronary vessels [2,11]. Sheng et al. [12] reported that circulating TMAO were associated with coronary atherosclerotic burden in patients with ST-segment elevation myocardial infarction. Furthermore, one study demonstrated for the first time a graded relation between TMAO levels and the risk of subsequent cardiovascular events in patients with recent prior ischemic stroke [13]. The aim of the present study was to investigate the serum levels of TMAO in Chinese patients with ischemic stroke.

## Materials and methods

### Patients and controls

From January 2016 to December 2018, consecutive patients with first-ever acute ischemic stroke admitted to Shanghai Jiao Tong University Affiliated Sixth People’s Hospital, China, were included. Acute ischemic stroke was identified by WHO recommendations (neurological deficit of cerebrovascular cause that persists beyond 24 h or is interrupted by death within 24 h) [14]. Magnetic resonance imaging (MRI) was used to validate the diagnosis within 24 h after the admission. Patients with the following criteria: (i) malignant tumor, metabolic syndrome and gastrointestinal diseases; (ii) symptoms onset more than 24 h at admission; (iii) liver and kidney function insufficiency; (iv) without informed consents; (v) other neurological diseases (such as Parkinson’s disease and Alzheimer’s disease) were excluded.

Two hundred and fifty-five age and gender-matched healthy volunteers from our Hospital Medical Examination Center were assigned to as the healthy control group. The median age of controls included in the present study was 65 (interquartile range (IQR), 57–71) years and 47% were women. In order to exclude the possibility that the controls could have any subclinical nervous system features, all control subjects were also clinically examined by the neurologist (H.L. and J.H.). Participants having any acute and chronic illness were excluded.

### Clinical data

Demographic data including age, sex, body mass index (BMI) and vascular risk factors, such as smoking habit, hypertension, diabetes mellitus, coronary heart disease, atrial fibrillation and a history of transient ischemic attack (TIA) were collected in the initial stage of the experiment. Meanwhile, patients who received pre-stroke therapy (antihypertensive and/or low-glucose drug) and acute treatment (tissue plasminogen activator-treated [TPA-T]) were also collected. Furthermore, the classification of stroke was achieved by using Trial of Org 10172 in Acute Stroke Treatment classification (TOAST), which distinguishes large-artery arteriosclerosis, cardioembolism, small-artery occlusion and other determined etiology [15].

Stroke severity was determined in each patient on admission by a neurologist (M.R.). The NIH Stroke Scale (NIHSS; scores range from 0 to 42, with greater scores indicating increasing severity) was the single judgement standard in the study for strokes severity [16]. Before any therapy, patients within 24 h on admission had their imaging examination using a Siemens Vision 3.0-T scanner (Siemens Medical Systems, Erlangen, Germany). Infarct volumes indicated by DWI were measured with version 3.0MIPAV software (NIH, Bethesda, MD) [17]. The infarct volume was calculated by a neurologist (M.R.) using the formula 0.5 × a × b × c (where a is the maximal longitudinal diameter, b is the maximal transverse diameter perpendicular to a, and c is the number of 10-mm slices containing infarct) [18]. Functional outcome of stroke patients was assessed at discharge according to the modified Rankin Scale (mRS) score (range: 0–6). Favorable functional outcome was defined as an mRS score of 0–2 points, while unfavorable outcome was defined as an mRS score of 3–6 points [18].

### TMAO measurement

Fasting blood samples were collected from each patient on the first morning after admission. Serum was stored at −80°C until analysis. Serum TMAO concentrations were measured by ultra-high-performance liquid chromatography-tandem mass spectrometry (UHPLC-MS/MS) using heated electrospray ionization (positive mode) and selected reaction monitoring as previously described [19]. Briefly, the UHPLC-MS/MS system consisted of an Accela autosampler, Accela UHPLC binary pump, and a TSQ Quantum Ultra triple quadruple mass spectrometer (Thermo Fisher Scientific, San Jose, CA). The assay described shows good inter- and intraday reproducibility (all CVs < 6.0%) and accuracy (>98.0% across low, mid and high values). Furthermore, high-sensitivity C-reactive protein (Hs-CRP), fasting blood glucose (FBG), triglyceride (TG), total cholesterol (TC) and homocysteine (HCY) were also measured using routine laboratory methods.

### Statistical analysis

Categorical variables were expressed as percentages and continuous variables were presented as medians (IQRs). Comparisons between groups were conducted using Mann–Whitney U test (continuous variables) or Chi-square test (categorical variables). Spearman’s rank correlation test was used for correlation exploration of laboratory parameters.

The stroke severity was dichotomized as minor (NIHSS ≤ 5) and moderate-to-high clinical severity (NIHSS > 6) [20]. Associations between TMAO and either clinical severity at admission were analyzed using logistic regression to obtain odds ratios (ORs) and 95% confidence intervals (CIs). Furthermore, associations between TMAO and functional outcome at discharge were assessed by logistic regression to obtain OR and 95% CI. Covariates included age, sex, BMI, stroke syndrome, stroke etiology, vascular risk factors, pre-stroke treatment, lesion volumes, and blood levels of TG, TC, HCY, Hs-CRP and FBG in the multivariable model. We tested the diagnostic value by calculating receiver operating characteristic (ROC) analysis. Thereby the area under the ROC curve (AUC) is a summary measure over criteria and cut-point choices.

At last, SPSS 22.0 (SPSS Inc., Chicago, IL, U.S.A.) and GraphPad Prism 5.0 were used for all statistical analyses, *P*<0.05 indicated the existence of statistical difference during the process of comparison.

## Results

### Baseline characteristics of study samples

From 345 patients with first-ever ischemic stroke (excluding 42 with onset of symptoms more than 24 h, 8 without informed consent, 6 with malignant tumor, 6 with renal insufficiency and 28 without MRI results), 255 patients were included into the study. The median age was 65 years (IQR, 57–71 years), and 136 (53.3%) were men. Thirty-one out of the 255 patients (12.2%) received TPA-T after admission.

### Main results

**Figure 1:**
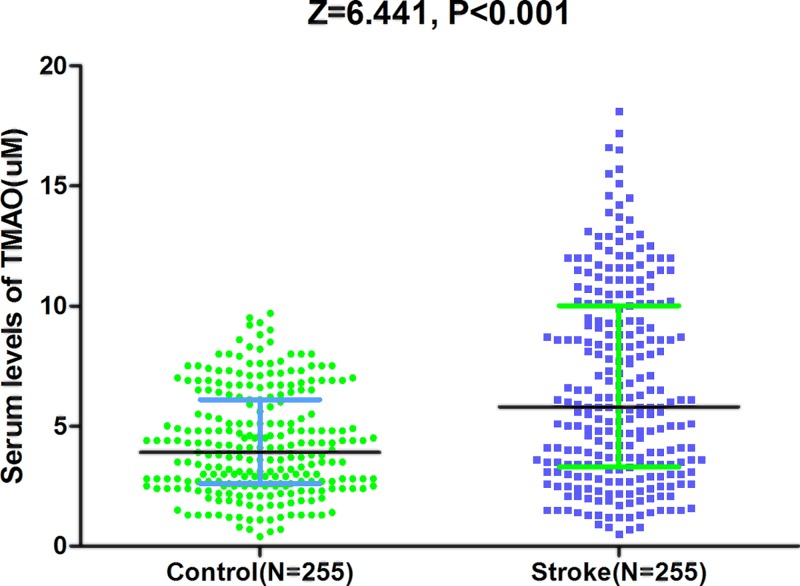
Distribution of serum levels of TMAO in ischemic stroke patients and in controls All data are medians and IQR. *P*-values refer to Mann–Whitney U tests for differences between groups.

**Figure 2:**
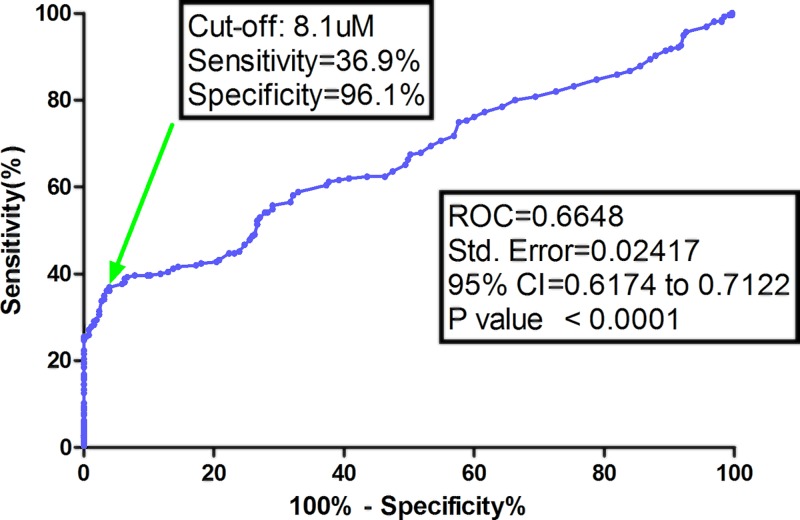
ROC curves were utilized to evaluate the accuracy of serum TMAO levels to predict ischemic stroke

The serum levels of TMAO ranged from 0.5 to 18.3 μM, with a median value of 5.8 (IQR, 3.3–10.0) μM, which was higher than in those controls (3.9; IQR, 2.6–6.1 μM), Figure 1. Based on the ROC curve, the optimal cut-off value of serum level of TMAO as an indicator for screening of stroke was estimated to be 8.1 μM, which yielded a sensitivity of 36.9% and a specificity of 96.1%, with the area under the curve at 0.665 (95% CI, 0.617–0.712), Figure 2.

By comparison, patients with cardioembolic strokes (*n*=53) had higher TMAO than those other stroke [6.6 (3.9–11.5) vs. 5.6 (IQR: 3.0–9.5) μM; *P*=0.018]. In addition, obvious positive correlations were presented between TMAO and Hs-CRP (r = 0.151, *P*=0.016), FBG (r = 0.145; *P*=0.025) and HCY (r = 0.186, *P*=0.003).

### TMAO and the severity of stroke

The characteristics of patients with mild stroke and those with moderate severe/stroke are shown in Table 1. Patients with severe stroke were significantly older, more frequently had atrial fibrillation, and had significantly higher serum levels of FBG, Hs-CRP and HCY.

**Table 1:** Characteristics of stroke patients with mild stroke and those with moderate-severe stroke^*^

Factors	Mild stroke	Moderate-severe	*P*
*n*	138	117	
Man-male	73 (52.9)	63 (53.9)	0.88
Age, median (IQR)	62 (53–68)	69 (63–74)	0.002
BMI, median (IQR)	25.2 (23.5–27.4)	25.5 (23.5–27.7)	0.75
Hypertension, *n* (%)	101 (73.1)	85 (72.6)	0.92
Diabetes mellitus, *n* (%)	37 (26.8)	30 (25.6)	0.62
Ischemic heart disease, *n* (%)	9 (6.5)	14 (12.0)	0.13
Atrial fibrillation, *n* (%)	11 (8.0)	20 (17.1)	0.03
TIA, n (%)	10 (7.2)	16 (13.7)	0.09
Smoking, *n* (%)	23 (16.7)	24 (20.5)	0.43
Pre-stroke treatment, *n* (%)			
Antihypertensive drug	103 (74.6)	87 (74.3)	0.96
Low-glucose drug	33 (23.9)	28 (23.9)	0.99
Blood testing, median (IQR)			
TG, mmol/l	1.20 (0.98–1.49)	1.29 (1.06–1.54)	0.19
TC, mmol/l	3.88 (3.23–5.05)	0.95 (3.29–5.15)	0.68
FBG, mmol/l	5.22 (4.76–6.15)	5.58 (4.96–6.49)	0.015
Hs-CRP, mg/l	0.43 (0.15–0.98)	0.69 (0.33–1.57)	<0.001
HCY, mol/l	15.5 (11.2–19.2)	17.8 (13.5–22.5)	0.002
TMAO, μM	4.1 (2.8–6.2)	9.1 (5.1–11.6)	<0.001
NIHSS score	4 (2–5)	10 (8–15)	<0.001
Stroke volume, ml	15.8 (10.2–20.5)	19.6 (13.5–27.8)	<0.001
Stroke subtype, *n* (%)			
LAA	30 (21.7)	20 (17.1)	0.35
SVO	32 (23.2)	23 (19.7)	0.50
CE	53 (38.4)	60 (51.3)	0.04
OT	23 (16.7)	14 (11.9)	0.29

* Moderate-to-high clinical severity is defined as NIHSS score >5. Variables are expressed as *n* (%) or median (IQR).

Abbreviations: CE, cardioembolic stroke; LAA, large artery atherosclerosis; OT, stroke of other determined etiology; SVO, small vessel occlusion.

**Figure 3:**
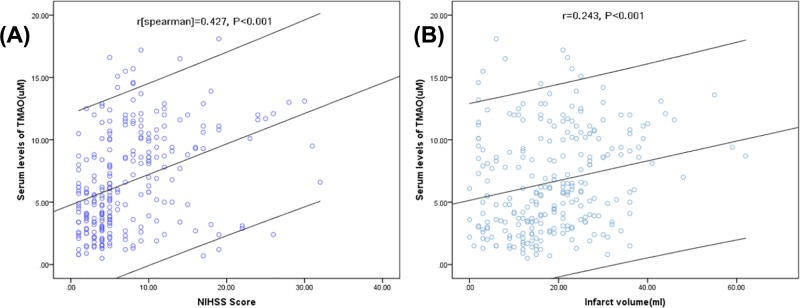
Correlation between serum TMAO level and other factors (**A**) Correlation between NIHSS score and serum TMAO level. (**B**) Correlation between infarct volume and serum TMAO level.

**Figure 4:**
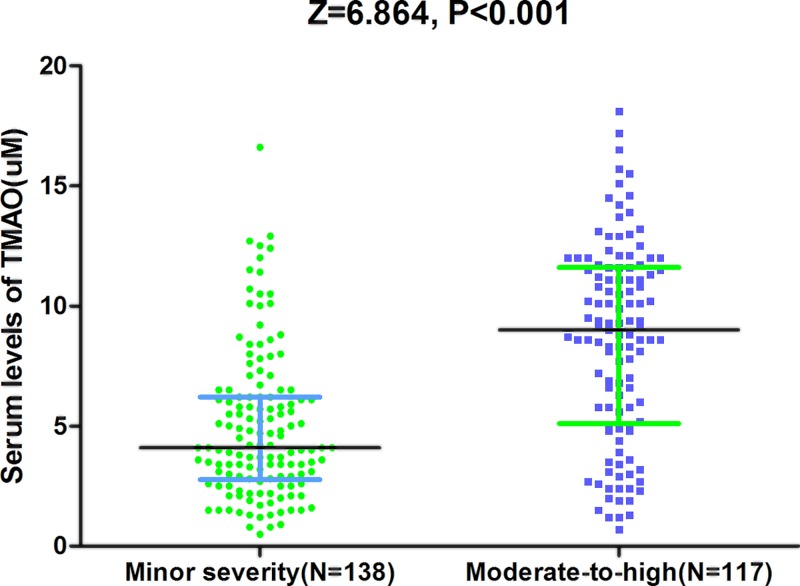
Distribution of serum levels of TMAO in patients with minor and moderate-to-high stroke The stroke severity was dichotomized as minor (NIHSS ≤ 5) and moderate-to-high clinical severity (NIHSS > 6). All data are medians and IQRs. *P*-values refer to Mann–Whitney U tests for differences between groups.

As a continuous variable, a positive correlation between NIHSS score and serum TMAO level (r = 0.427; *P*<0.001) was found, Figure 3A. In the median regression model, NIHSS score of 3.80 point was measured to be increased significantly for every 1-point increase in TMAO (95% CI, 3.20–4.60; *P*<0.001), amount to a median increase in approximately 1 point in NIHSS score for every 0.263-point increase in TMAO. At admission, there were 138 strokes were defined as minor stroke (54.1%, NIHSS < 6). The median level of TMAO in those patients were significantly lower than in those moderate-to-high stroke (4.1 μM [IQR, 2.8–6.2] vs. 9.1 μM [5.1–11.0], Z = 6.864; *P*<0.001; Figure 4), with an obvious median difference of 5 by statistical analysis (95% CI, 3.2–8.5; *P*<0.01). In univariate models, the unadjusted risk of moderate-to-high stroke was increased by 31% (OR = 1.31 [95% CI: 1.21–1.42], *P*<0.001), when TMAO was increased by each 1 μM. The following variables were put into multivariable logistic regression model: age, sex, BMI, stroke syndrome, stroke etiology, vascular risk factors, pre-stroke treatment, lesion volumes, and blood levels of TG, TC, HCY, Hs-CRP and FBG. The results revealed that TMAO was still associated with an increased risk with moderate-to-high stroke (OR = 1.22; 95% CI = 1.08–1.32; *P*<0.001), which was described in Table 2. In addition, as shown in the Table 2, age, infarct volume, CRP and HCY also independently predicted severe stroke unlike other factors.

**Table 2:** Multivariate analysis of predictors of moderate-to-high clinical severity^*^

Predictors	OR	95% CI	*P*
TMAO (per unit increase)	1.22	1.08–1.32	<0.001
Age (per unit increase)	1.25	1.03–1.42	0.013
Infarct volume (per unit increase)	1.15	1.03–1.30	0.009
Atrial fibrillation (Yes vs. No)	1.56	1.02–2.43	0.043
Stroke etiology (CE vs. other)	1.68	0.89–2.95	0.13
FBG (per unit increase)	1.68	0.95–3.16	0.092
CRP (per unit increase)	2.05	1.39–3.44	0.021
HCY (per unit increase)	1.09	1.02–1.22	0.034

* Multivariable model included all of the following variables: age, sex, BMI, stroke syndrome, stroke etiology, vascular risk factors, pre-stroke treatment, lesion volumes and blood levels of TG, TC, HCY, Hs-CRP and FBG; moderate-to-high clinical severity is defined as NIHSS score > 5.

**Figure 5:**
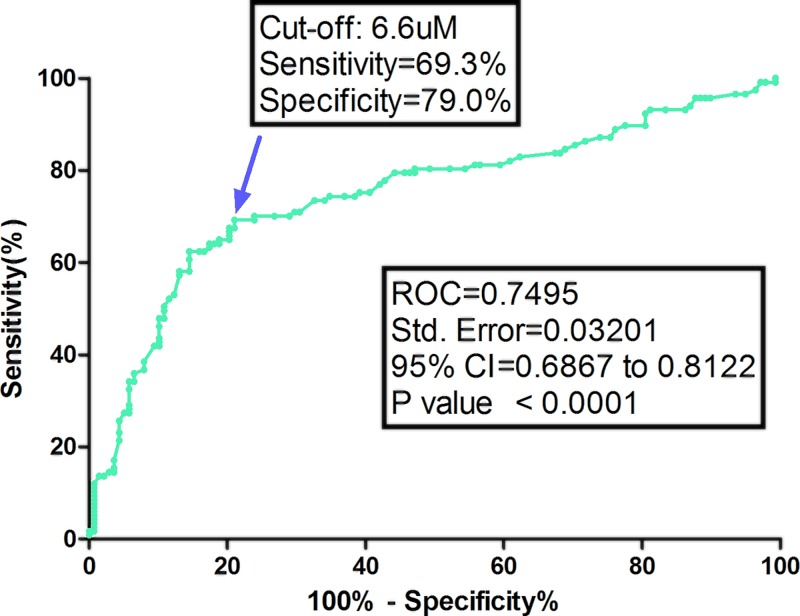
ROC curves were utilized to evaluate the accuracy of serum TMAO levels to predict moderate-to-high clinical severity The stroke severity was dichotomized as minor (NIHSS ≤ 5) and moderate-to-high clinical severity (NIHSS > 6).

Based on the ROC curve, the optimal cut-off value of serum level of TMAO as an indicator for screening of moderate-to-high stroke was estimated to be 6.6 μM, which yielded a sensitivity of 69.3% and a specificity of 79.0%, with the area under the curve at 0.750 (95% CI, 0.687–0.812), Figure 5.

Median DWI infarct volume was 18.0 ml (IQR, 11.0–25.0) in all patients. DWI infarct volume was much higher in patients with moderate-to-high stroke than those with minor [19.6 (13.5–27.8) *vs.* 15.8 (10.2–20.5) ml; *P*<0.001)], Table 1. As a continuous variable, a positive correlation between infarct volume and serum TMAO level (r = 0.243; *P*<0.001) was reported, Figure 3B. At the same time, increased infarct volume of 12.5 point was also observed for every 1-point increase in TMAO (95% CI, 9.6–15.3; *P*<0.001), amount to a median increase in infarct volume of approximately 1 point for every 0.08-point increase in TMAO.

### TMAO and stroke functional outcome

At discharge, 175 patients (68.6%) had favorable outcome whereas 80 patients (31.4%) had unfavorable outcome. In the latter group, the median TMAO level was higher than that observed in patients with favorable outcome (9.0 [IQR: 5.5–11.5] μM vs. 4.8 (2.7–8.5) μM; *P*<0.001). In multivariate logistic regression analysis (adjusted for age, NIHSS, CRP, HCY and other factors), a higher level of TMAO was associated with a higher risk of unfavorable outcome, and the risk increased by 14% (OR = 1.14; 95% CI = 1.04–1.23; *P*<0.001) for each 1 μM increase in serum level of TMAO.

## Discussion

Accumulating evidence suggests that TMAO, a gut microbial metabolite, is implicated in the pathogenesis of many cardiovascular and cerebrovascular diseases [21–23], and has been shown to be atherogenic [2,9,10]. In this study, the data suggested that (i) the patients with ischemic stroke had higher levels of TMAO than those normal controls, suggesting that higher TMAO levels were associated with increased risk of first stroke; (ii) patients with higher levels of TMAO had worse neurological deficit (defined by NIHSS score and infarct volume).

Consistent with our findings, one study reported that TMAO was an independent predictor of ischemic stroke, which could potentially refine stroke stratification in patients with atrial fibrillation [24], while another study confirmed that higher TMAO levels were associated with increased risk of first stroke in hypertensive patients [25]. In addition, Ha et al. [26] reported that serum TMAO levels were associated with infarction volume in mice ischemic stroke model.

Furthermore, the role of TMAO in atherosclerosis had been proposed in previous studies [9–11]. One study found that fasting plasma TMAO levels were an independent predictor of a high atherosclerotic burden in patients with coronary artery disease (CAD) [27], while another study reported that increased TMAO concentrations correlate with coronary atherosclerosis burden and may associate with long-term mortality in patients with CKD undergoing coronary angiography [3]. A meta-analysis had demonstrated the positive dose-dependent association between TMAO plasma levels and increased cardiovascular risk and mortality [28], while another meta-analysis suggested that higher circulating TMAO may independently predict the risk of subsequent cardiovascular events (HR = 1.23, 95% CI: 1.07–1.42, *I^2^* = 31.4%) [29].

Despite these initial findings, the association between TMAO and cardiovascular disease were not consistent in all population studies to date. One study in North Americans including mixed-race healthy volunteers (33–45 years) showed that plasma TMAO levels were not associated with incident coronary artery calcification or coronary artery intimal-medial wall thickness over a 10-year follow-up period [30]. Similarly, another study including 339 patients undergoing coronary angiography found no association between plasma TMAO and prevalent CAD or incident events over an 8-year follow-up period [31]. These conflicting data linking TMAO with cardiovascular disease risk might have resulted from differences in study methodology, diet, ethnicity, environment and prevalent patterns of gut microbiome composition in study subjects.

In ischemic stroke patients, gut dysbiosis and increased bacterial counts of *Lactobacillus ruminis* subgroup in the fecal gut microbiota was associated with elevated systemic inflammation and altered metabolism [32]. Increased gut permeability can promote inflammatory responses, whereas systemic inflammation can increase gut permeability [33]. Bacterial and endotoxin translocation to the blood stream, increased proinflammatory cytokines, and systemic inflammation can induce or exacerbate cardiac dysfunction [34,35]. Altered TMAO levels has been associated with impaired cardiac function, heart attack, and heart failure [10,35].

The adverse effects of TMAO on cardiovascular function might be associated with multiple mechanisms. (i) TMAO promotes atherosclerosis, probably through an increased expression of macrophage scavenger receptors and formation of foam cells in the artery wall [2]. (ii) High levels of TMAO promote endothelial dysfunction, exacerbate platelet reactivity and enhance thrombosis, affect lipid metabolism and inflammatory response, underlying the importance of this molecule in the cardiovascular system [10,36,37]. Endothelial dysfunction, in part, resulting from excessive production of reactive oxygen species (ROS) and inflammation, is an important mechanism of cerebrovascular damage [38]. (iii) Recent studies demonstrated that TMAO promotes vascular inflammation and oxidative stress, inhibits eNOS expression and activity and reduces NO production, which are associated with endothelial dysfunction and atherosclerosis [2,37,39]. (iv) One study supported the notion that TMAO-related increase in proinflammatory monocytes may add to elevated cardiovascular risk of patients with increased TMAO levels [13]. (v) TMAO promoted vascular inflammation by activating the nucleotide‐binding oligomerization domain-like receptor family pyrin domain-containing 3 (NLRP3) inflammasome, and the NLRP3 inflammasome activation in part was mediated through inhibition of the sirtuin 3 (SIRT3)‐superoxide dismutase 2 (SOD2)-mitochondrial ROS signaling pathway [40].

Some limitations in the present study should be considered. (i) A lack of ethnic diversity (all Chinese) which may affect the generalizability of our findings to other populations. (ii) We did not have any information on gastrointestinal symptoms or antibiotic use before blood sampling. (iii) We could not exclude the potential for dietary intake of choline or TMAO (e.g. large consumption of some fish species) within 24 h before blood sampling. (iv) Serum level of TMAO was measured one time at admission, and we did not test TMAO in other time-point. (v) Whether TMAO is pathogenically involved in the development of stroke would be a more interesting topic, nevertheless, the observational study does not allow advancing any cause and effect relationships. In addition, the role of TMAO in stroke outcome was not explored.

## Conclusions

In summary, higher TMAO levels were associated with increased risk of first stroke and worse neurological deficit in Chinese patients, suggesting chronic dietary exposures that increase TMAO appear to directly contribute to ischemic stroke.

## Ethical statement

An approval of the study was received from the Ethics Committee of the Shanghai Jiao Tong University Affiliated Sixth People’s Hospital, China. All participants or their relatives were informed about the study protocol, and their written informed consents were obtained before participating in the study.

## Ethics, consent and permissions

Written informed consents were obtained from all patients; and, the present study conformed to the principles of the Declaration of Helsinki was approved by the Investigational Review Board of the Shanghai Jiaotong University.

## Data Availability

Please contact author for data requests.

## Abbreviations

BMI: body mass index
CAD: coronary artery disease
CI: confidence interval
CVS: coefficients of variations
DWI: diffusion weighted imaging
ENOS: endothelial nitric oxidesynthase
FBG: fasting blood glucose
HCY: homocysteine
Hs-CRP: high-sensitivity C-reactive protein
IQR: interquartile range
MRI: magnetic resonance imaging
mRS: modified Rankin Scale
NIHSS: NIH Stroke Scale
NLRP3: nucleotide‐binding oligomerization domain-like receptor family pyrin domain-containing 3
OR: odds ratio
ROC: receiver operating characteristic
ROS: reactive oxygen species
TC: total cholesterol
TG: triglyceride
TMAO: Trimethylamine-N-oxide
TPA-T: tissue plasminogen activator-treated
UHPLC-MS/MS: ultra-high-performance liquid chromatography-tandem mass spectrometry
